# Proceedings of the American Dental Association

**Published:** 1870-09

**Authors:** 


### ARTICLE II.

### *Proceedings of the American Dental Association.*

The tenth annual meeting of the American Dental Association convened in the hall of the House of Representatives, at Nashville, Tennessee, on Tuesday August 2, 1870

At 10 A. M., the President, Prof. Homer Judd, of St. Louis, called the body to order, and the session was opened with prayer by Rev. T. O. Summers, D. D.

Dr. W. H. Morgan, of Tennessee, the Chairman of the Committee of Arrangements, then welcomed the association in a pleasing address.

Prof. C. K. Winston, M. D., by authority of the faculty of the University and the medical profession of Nashville, delivered an eloquent speech of welcome. He praised the perfection to which the art and science of dentistry had been brought, and he was proud to claim full kinship with them as professional brethren.

Dr. C. P. Baird, President of the Tennessee State Dental Association, delivered an address in behalf of that body.

The roll was then called, and over sixty members were found to be in attendance.

On motion of Dr. E. A. Bogue, of New York City, the reading of the minutes was dispensed with.

It was resolved, on motion of Prof. L. D. Sheppard, of Boston, that all resident dentists, physicians, lawyers, and clergymen be invited to take seats at the meeting.

The members of the Committee on Prize Essays were all absent, and on motion, the chair appointed Prof. J. Taft, of Cincinnatti, Ohio, Drs. W. O. Kulp, of Muscatine, Iowa, D. S. Dickerman, of Boston, Mass. and J. R. Walker, of New Orleans, La.

The Committee on Dental Physiology were also absent, and Prof. S. P. Cutler, of New Orleans, La., Drs. George H. Cushing and J. N. Crouse, of Chicago, Ill., were appointed.

Of the Committee on Voluntary Essays, Dr. C. D. Cook, of Brooklyn N. Y. being present, became chairman. Drs. S. H. McCall, of Binghamton, N. Y., and J. Fouche, of Knoxville, Tenn., were added.

Dr. H. E. Peebles of St. Louis, Mo., moved that a committee of three be appointed to collect information of the whereabouts of the bound volumes of the Transactions, from the several committees having them in charge, and report to the association upon the propriety and practicability of disposing of them to persons not members.

Profs. Sheppard, Taft, and Dr. M. S. Dean, of Chicago, Ill., were appointed by the chair.

Dr. Bogue moved to adopt informally the printed programme furnished by the committee of arrangements. Carried.

Drs. Walker, A. C. Ford of Atlanta, Ga., and Peebles were appointed the Committee on Dental Instruments and Appliances

The reports of the committees not being ready, a motion to adjourn was carried at 11.05 A. M.

FIRST DAY.—*Afternoon Session.*

Called to order at 3 P. M.

After the reading of the minutes, the Committee of arrangements reported the credentials of the members correct. Adopted.

Prof. W. H. Atkinson, of New York, read the report of the Committee on Dental Pathology and Surgery.

Other written communications on this subject were called.

Dr. McCall, of the Committee on Voluntary Essays, reported that he had a paper to read, but that it was not written by a member of this association.

A motion was made and carried that it be read.

Dr. Morgan moved a reconsideration of the vote, as he thought it would be a bad precedent to allow parties not members to occupy the time of the association.

Prof. Atkinson hoped the society would hear the paper.

Dr. Crouse opposed it, as quacks could thus thrust themselves into notice and have worthless matter read.

Dr. S. J. Cobb, of Nashville thought the danger magnified.

Prof. Bogue favored the reading, and did not approve of exclusion.

Prof. Sheppard thought the writer of the article had the dental journals and local societies to offer the communication to, but that there are too many who do not take interest enough in either to support them.

Prof. Stellwagen said he came here to leaın, and he cared not from what source his fund of knowledge was increased, provided it was truth; he could not suppose it would injure any one. What are committes for, if not to be the judges of the propriety of bringing papers offered to them before this body? If the three gentlemen who composed the committee had read the paper and felt that it was worth repeating, he could not do less than listen. He knew the writer, and believed he was connected with a local society, and was, if he is not now, an emeritus professor tn a dental college.

Prof. Taft, of Cincinnati. and Dr. W.W. A!lport of Chicago followed in opposition to it as a precedent. The debate was closed, and the original motion lost.

Prof. Atkinson then proceeded with remarks upon pathology. He had felt impressed with the utter want of certainty as to what is health and what is disease.

By means of a diagram of the universe, he attempted to show how, as matter advanced in the scale, it took up new attributes, and how, with animals, their different senses gave different functions and capabilities of enjoyment.

He proposed the use of diluted sulphuric acid, one or more drops to thirty of water, for treatment of caries of bone, applying it carefully upon a thread of lint wound round an instrument or wire, and passed into the fistulæ, which might be enlarged by plugging with tents, and allowing the moisture to swell the plug and thus make an opening even large enough to look into the bone or pass a finger in. He claimed that the action of acid dissolved the mineral portion of the diseased bone, aiding its removal by washing, and yet that it would not injure, but rather stimulate the healthy structure to throw out granulations.

Prof. Cutler proposed to follow Prof. Atkinson. Phosphate of lime is a tribasic salt. We have been told that sulphuric acid, hydrochloric acid, etc. form a solution. But this acid takes out one equivalent of the lime, and it becomes an acid salt superphosphate. Hydrochloric acid produces a soluble salt, so does vinegar, etc.; but the sulphuric acid makes a comparatively insoluble salt. When chloride of zinc is brought in contact with lime, the zinc is turned loose as oxide, and the chlorine takes up the atom calcium, forming chloride of calcium.

Necrosis, he said, meant dead bone. Bone is the lowest tissue in the scale of life, except the teeth, and they are between the animal and crystalline, particularly the enamel; they are endowed with the most sensitive nerves, not excepting the eye or ear. If the dentinal tubuli project into the enamel, the substance is very sensitive.

Adjourned.

### Second Day.—*Morning Session.*

Meeting called to order at 10 A. M.; seventy members present.

Prayer was made by Rev. Dr. Young.

Reading of minutes postponed until the afternoon.

The resignation of Dr. George Watt, of Cincinnati, was read and accepted.

The following resolutions, by Dr. Peebles were adopted.

*Whereas*, It is patent to our profession that great ignorance obtains among American mothers regarding the eruption and development of the teeth of their children; and *whereas*, such ignorance is one of the main causes of the loss of many teeth, and especially so of the six year old molars; and, *whereas*, to remedy this great evil will require a thorough and early course of rudimental instuction; therefore

*Resolved*, That a committee of three be appointed by the chair, whose duty it shall be to correspond with the publishers of American school books, and ascertain if some plan can be devised to have short, plain statements inserted of the number, name, form, and arrangement of the several teeth in the deciduous and also in the permanent set.

*Resolved*, That the committee be and is hereby authorised to make all the necessary arrangements with the said publishers of school books to carry out fully the ideas embraced in the preamble and first resolution, and report at the next meeting of this association.

Drs. Peebles, Fouche, and S. W. Dennis of San Francisco, were appointed as a committee for the carrying out of the resolution. Drs. Kulp and Cobb were added to the committee.

The discussion of "Pathology" was declared in order, and Prof. Cutler continued his remarks of the previous evening.

He said it had been stated that necrosed alveolus could be restored; he thought, if done, it was an exception to the general law of nature. He then described the condition of the tooth, and the relations between it and alveolus by periodentium or pericementum, when this membrane is left bare or denuded by violence, action of arsenic, or cobalt, or any cause. The cementum is like true bone, having all the characteristics; but he would call it super-bone, having more than a bone, for passing through it are the minute fibrillæ from the nerve pulp of the tooth. When denuded, it is extremely sensitive to any foreign body; but bone is not; even in amputation, the pain is only when cutting through the marrow. Now, when exposed, the nerve fibrils of the cementum give extreme pain, and the condition is one of hyperæsthesia. Even when denuded and dry, this is the case. To furnish a new matrix and restore bone will take two years; but we cannot restore the membrane of the tooth upon which to build, or with which the periosteum of the bone unites; no provision has been made by nature; the dentine has no vascular system. When roots of teeth are united, it is always by the cementum. The alveolus in a case he recently had was dead, dry, and necrosed; he broke it out, and the pericementum was dead; but nature had made no attempt to reproduce this pericementum. There is consequently no means of attaching the pericementum to the cement of the tooth. In the tooth we have a neural system projecting beyond the hæmal; the nutrition is extravascular, as throughout the body. Bone is the only tissue that is reproduced; but others, when seperated, are united by connective tissue, as cicatrix in muscle. At the point of a fang we may have an abscess, which may be cured and the space filled by cartilage.

Dr. Walker. All are familiar with the fact of the re-formation of the alveolar process where extraction has taken place. Why should we not have the same where the tooth remains.

Prof. Judd arose to take exceptions to some of the points of the last speaker. He would do this to guard from assumptions in the study of the laws of life unless proven by actual facts.

The periosteum, the gentleman claimed, was destroyed; he did not know of any experimets or physiological laws that would demonstrate this periosteum to be dead. Logical deductions lead often to monstrous conclusions. The basis of periosteal structure and pericementum is the same. Beale traces one running into the other. Where there is periosteum, we have the elements to produce new bone, and even the marrow will make periosteum. These facts have been actually demonstrated, and he could not see why it should not be so with pericementum. In the Odontological Society, we have the fact reported that teeth had been extracted, periosteum scraped off, then replaced, and growing fast and firm. After experiments upon producing bone to fill up cleft palate, by dissection of the periosteum, and bringing these flaps together, bone has been found to form under the cicatrix.

Prof. Cutler explained that he had fallen back upon facts, and he had never seen or heard of a case where the pericementum had been reproduced.

Dr. J. G. Willis called attention to the remark that foreign substances could only be gotten rid of by expulsion or encysting. Recently experiments have been made that prove that necrosed bone may be removed from its position without ulceration. The extravasation of blood in brain, or a musket-ball projected into muscle, are familiar instances of encysting. Prof. Lyster gave a case where a sphacelus of the tibia had been removed by the use of carbolic acid without any ulceration.

Dr. Walker. One of the most important matters to call attention to is the hydra-deaded monster, the new disease in the mouth from the use of rubber plates. He had met it before the war, but gave up practice then, and upon returning to practice again found trouble In every case examined he found disease. There is first thickening of mucous membrane, then sores appear, looking often like a piece of half spoiled beef cut across the grain. He found the processes of the jaw bones often absorbed as a result of this disease.

Dr. E. A. Herman asked if the doctor saw it in any mouths where other plates were used.

Dr. Walker. No.

Dr. S. B. Palmer. Is the non-conducting property of the vulcanite the cause?

Dr. Walker thought the non-conductibility of the plates had little to do with it; he thought the work due to articles used in the manufacture, such as mercury, etc.

Dr. John Allen, of New York, spoke of a lady who had a set of teeth that caused sloughing of soft tissues and necrosis of palate. He analyzed a portion of the plate for the sake of determining what it contained. Her usefulness is entirely destroyed; her occupation, that of a school teacher, has gone. When last heard from, all hope was nearly gone. A continuous salivation was going on. His experience was that in nearly every case where rubber is worn he had seen this soreness. His convictions, aside from any preference for other substances, are that it should not be used.

Dr. Morgan had seen few mouths where there was not a disease if artificial work was worn. Nature had not intended false roofs to be worn. He said Dr. Allport had reported one case where platina had been worn and given no trouble. He saw a case here in Tennessee, where a lady had trouble from wearing a plate of coined gold. Idiosyncrasies are discovered where false dentures are worn.

Another case cited was that of a lady wearing an eighteen carat gold plate, who had raw sores in her mouth; this was cured by wearing vulcanite. This does not prove any rule, however, for there are too many exceptions.

Dr. Atkinson. Did the patient wear the plate during the night?

Dr. Morgan. She did not, and often rested the mouth during the day; special regard was paid to cleanliness.

Dr. W. O. Kulp, of Iowa, said: Since this poison cry against the rubber base,I commenced a series of investigations, and, by the aid of friends, eleven hundred cases have been examined—seven hundred where red rubber was worn, one hundred and forty gold plates, two hundred and thirty silver plates, ten platina, and twenty of other bases, such as cheoplastic, tin, alumiuum, etc. In proportion, we found fewer mouths in a diseased condition where rubber was worn than where silver; about as many in proportion where gold plate was used. Tin and cheoplastic uniformly produced a diseased condition of the mucous membrane; where aluminum, having teeth attached, with some other compound, was worn, the diseased condition of the membrane extended all over the mouth. Uniformly the testimony was that rubber was worn with more ease than other substance, except from its extra thickness. The cleanest mouths, *i.e.* the most free from disease, were those where aluminnm was used, with teeth attached to plate with black rubber. My investigations illustrate that it is an abnormal condition to wear a covering over the roof of the mouth, and that any plate is capable of producing a diseased condition of the mouth if worn constantly and not kept perfectly clean. The result of these investigations ought to make us redouble our efforts to reach the blessed time when no more artificial teeth are demanded, but when all the natural teeth will be saved.

Dr. W. A. Jones wished to state one matter—that in most of these cases mercury had been found present in the system. All metals have an affinity for mercury; probably platinum has the least. The worst cases he had seen were from wearing gold plates. He had seen less trouble from vulcanite than anything, except Dr. Allen's continuous gum work. He had a patient, a physician, who could not wear a gold plate but a few times. Every week or so, by taking out the plate, mercury was found upon it, which, being driven off by heat, he could wear it. A black rubber plate vulcanized very highly, was tried with success in this gentleman's mouth.

Dr. C. C. Carroll. Are we not off the subject of pathology?

Prof. Atkinson wished to ask if it is mercury, the metal or sulphuret of mercury, secreted in the system, which causes the trouble.

Dr. Jones could not answer whether it was the sulphuret or metallic mercury.

Dr. Allen. With reference to the trouble from platinum plate, the case mentioned was the only one he knew of where a plate properly fitting had given trouble. The swaging of the plate in base metal will account, he thinks, for the surface being covered with base metal. He takes special pains not to let the metal dies come in contact with the plate, putting thin tissue-paper between the plate and dies. He had found that the more continuously persons wear a plate, the better it fits, on that account he wears his plate all the time, even while sleeping.

Dr. J. P. Holmes. Does not the atmospheric chamber cause trouble?

Dr. Allen. Yes, in ninety nine cases in the hundred it is the unequal pressnre. A deep air-chamber should not be used, as it draws down the mucous membrane.

Dr. Crouse. There certainly would be no more pressure in a deep air-chamber than in a shallow one, only that it would be more to fill up. As to the idea of mercury, quinine, or calomel in the system, it is all a notion. It is erroneous, and out of the question. He thought rubber produced more trouble than other substances, but this was due to the non-conductibility of the material. He had a case of granulated and thickened membrane under a vulcanite plate. The periosteum was diseased; teeth loosened. After removal of the plate for two weeks the teeth grew solid in their sockets. The plate was held in position by clasps; no air-pressure. It had been worn three years. He replaced this with a gold plate, and it gave comfort.

Dr. Walker. With regard to vulcanizing, he had found where highly vulcanized it was worse, and where the plate was close to the teeth or resting on them, he found them losing the lime salts more rapidly than when other materials were worn. With regard to cleanliness, where he had seen this trouble it was in the mouths of persons most careful and particular. In one case he had seen a gentleman whose mouth and throat were sore for some time after wearing a rubber plate. A physician of his city had had a relaxation of the palate from wearing a small plate.

Dr. J. W. Moffit, of Harrisburg, Pa., for fourteen years had been using plates of different materials. The friction was generally the cause of the disease, but in rubber plates he found an additional cause. One case was cited where a lady had her mouth covered with small ulcers. On laying aside the plate she recovered, and on the resumption of the plate the trouble returned.

Dr. I. Forbes of St. Louis. What was the temperament?

Dr. Walker had seen all temperaments affected. One case mentioned was nervo-sanguine.

Dr. Forbes. Was the nervous the predominant temperment?

Dr. Walker. No.

Dr. Forbes. Platinum is good conductor of electricity—better than gold, and he thought as good as copper. His humble judgement is that platinum plates will excite inflammation in mucous membrane sooner than any other. He had a patient who had worn a plate of platinum which gave trouble, but a gold plate gave none. Fillings of different materials will give trouble iu the mouths of nervous people. Rubber he denied being a good conductor of heat. It had been lately demonstrated that the electricity of the mouth differed as the nervous system predominated; that fillings of different materials made batteries, and around one pole was found decay. The difference in the purity of metals he could not say would produce a current, but a tin or amalgam filling with gold would. Aluminum being a better conductor of electricity, would be a better filling material. With regard to scraping the periosteum and returning teeth, he had, when a young man, taken out and filled a wisdom tooth for a lady. It had lasted five or six years, and then he lost sight of the patient by her going away from the city. He had also extracted a bicuspid, and syringing out the cavity he replaced it. The tooth remained in the mouth for years, indeed until the death of the patient.

Dr. Walker said that, without any exception, he had found the disease in all cases and with all temperaments.

Dr. Peebles is of the opinion that the trouble in partial sets is mainly owing to the fact that in this kind of work they often fit up the plate to the necks of the teeth. This is to secure firmness and fixedness. When using gold we are not so particular, as the suction of the gold used is trusted to. He had put in a case of rubber where a lady wearing it was very ill for fifteen days after its use. A few hours after her arrival at her boarding-house, after having the plate put in her mouth, she was taken violently ill, vomiting a greenish fluid. He readjusted the plates, and called upon a physician who led her to think she was poisoned, and they agreed to keep her in town for another trial. The plates had been vuicanized very hard. New ones were made not so hard, and in three hours she was again taken sick as before. He finally made gold plates, which gave no trouble.

Prof. Judd. This question is one of very great interest. He would fail in his duty to his profession and his conscience if he did not speak. He had no axes to grind, and no fight for or with anything—he only wanted truth. Let us look calmly at the subject. Should we not look to it that we do not produce worse diseases than we are called upon to relieve? He wished to call attention to the fact that there are various diseases caused by plates. Let us describe them as we see them,—one from mechanical irritation, another from poisoning, and another from suction or clogging the pores. No one in such a climate as he lives in could fail, if he closely examines, to find a disease from the use of plates. He had seen the mouth covered with spongy masses, filling up the arch with a soft tissue, such as he had never seen anywhere else, distinctly marking a different disease,—a purple-colored, strawberry-like looking mucous membrane. He could quote case after case where he had seen necrosis of the vomer, superior maxillary bones, etc. etc. These were generally in strumous patients.

What are the plates made up of 40 per cent. rubber, 24 per cent. sulphur, and 36 per cent. of mercury? call them by their right names,—mercury and sulphur plates. It may be said that the material is insoluble; but so is arsenic, and who would put in an arsenical plate? He would not feel as if doing his duty if he recommended this compound of mercury, sulphur, and caoutchouc.

Prof. Atkinson. I am glad of all said, but am sorry to see us spending the time in the details of practical life. Let us try to understand principles, and be done with cradle songs. Men who have been pricked to the heart for love of truth must talk. I am not going to worship mercury or Hygeia,—she can take care of herself. Let us pour out whole mouthfuls of seed-thoughts. What is the cause of necrosis? Can mercury cause it? I challenge the world to prove it. I

hold that until made a binary it cannot enter the system, but may be between the cells as a simple element entangled in the tissues.

Let us get at truth only. Anybody who has shot squirrels goes to the hickories. Any one who wants to shoot mercury must not go into the bones, but go along the alveolar processes. It dont dissolve the tissue. What then, where a huge necrosis is found? It is syphilis; he would chase it out like a squirrel, and tell the length of its tail. Syphilis is the cause; it is the result of transgression of connubial law. Treat the case as if it were syphilis, and, if proper means are used, if you are not successful, send the bill to me, and I will take care of it financially.

In primary syphilis, extirpate the first portion of poison before it has time to telegraph to the whole body. In secondary, use biniodide of mercury and potash. In tertiary, just go it on iodide of sodium. Circulation, respiration, and innervation mean digestion—that is, of the pabulum on which the tissues feed. After this comes the assimulation. Cleanliness would cure the whole thing, if we knew what it meant, or how to go back and clean every tissue.

Extract no more teeth; mutilate no more people. Every time we do we lay the bone liable to disease, and the teeth are not there to warn us of it. Outside of zymotic diseases there is none other than syphilis or its effect, resulting from a poison that perhaps may have been introduced into the system of ancestors and handed down. Enamel, dentine, cementum, and bone are all under crystallosophy, and for this he dissolves out the mineral portion and leaves only the animal portion to bring about the cure under the ghosts of their corpuscles.

If gentlemen will say that rubber was the exciting cause for the disease, don't for five, ten, thirty, fifty, or one hundred and fifty dollars, cut your souls all into scars, so that when you come to heaven's gate Peter won't know you.

Committee on Dental Ethics filled by Prof. J. S. Knapp, of New Orleans.

A letter from Prof. J.B. Lindsey, M. D. of the Faculty of the University of Nashville, was read, inviting the members of the American Dental Association to visit the museum. Half-past two o'clock on Thursday, was the hour decided upon for the visit, and the invitation was accepted.

SECOND DAY.—*Afternoon Session.*

Minutes read and approved.

The Chair appointed as Committee on Mechanical Dentistry, Drs. Wright and T. T. Moore, of Columbus, S. C.

Dr. Cushing called attention to the marked difference in the experience of persons from various sections of the country, seeming to show something to be due to the climatic influence. In the case spoken of by Dr. Crouse, he had noticed that the mucous membrane, where the plate was not worn, was very healthy in appearance. He had not seen trouble from either gold or platinum plates.

Dr. Taft. This is an exceedingly broad subject, as indicated by the range of the discussion. He preferred that they be confined to general principles, rather than to mentioning the minutiæ of cases. Every one who feels an interest in maintaining these bodies in good condition will see the necessity that this discussion must be kept within bounds. It is more pleasant to contemplate the body in healthy working condition than in disease. Perhaps we, in our anxiety to contend with and vanquish disease, go too far; nevertheless we meet with disease to be remedied and overcome. He had been much interested and instructed in the discussion. The causes of these affections have been referred to, but not as fully as we could wish. Disturbances result from various causes, and doubtless all see how important it is to study for themselves, so as to become familiar with idiosyncrasies and differences of constitution. It is imperative that all should understand the laws of life.

He said Prof. Atkinson seemed to consider all disease due to the introduction of inimical material into the system. No doubt with some but a small amount will, if introduced, produce trouble. The treatment of wasted alveolar process and diseased bone—namely, the introduction of properly diluted sulphuric acid, so as to dissolve and permit the diseased bone to be washed away—of course requires care and knowledge. Other acids were mentioned by Prof. Cutler; but he thought the sulphuric acid would act without injuring the living part beyond the diseased. Nitric acid or glacial acetic acid would not operate so well. The method of applying was interesting, opening up the cavity and introducing the tent, making but small disturbance among the soft parts around. This requires great care in its use.

For abscesses he had used the tangle tent, a woody, fibrous structure, which, on being introduced, expands from two to four times its diameter, opening up the fistula so as to admit of treatment. There are various causes to be taken into account; their character should be well understood, and the character of the structure should be considered. The structure has its individuality, like the face or body,—one is of strong the other feeble vitality. It has been said that all are alike in this, and that the difference of susceptibility is due to the difference of the working of the organs. We should study all these differences, and although there is much that cannot be readily found there is that which is readily seen upon examination. Some may be able to diagnose without being able to convey to one another how it is done. We must study nature and physiology, and governing laws. Sometimes he thinks we live to fight this enemy of ours, but he would say that it is important to prevent the entrance of disease.

Prof. Cutler would ask what constitutes pathology, if it is not deviation from physiology. When the dentist extracts teeth, is he not bringing about a pathological condition? When he converts the pathology into physiology, then he is a surgeon; but the other operation is that butchery of extracting teeth as performed by quacks, who replace them with their miserable subsitutes. He thought it the duty of mothers, of teachers of day and Sunday-schools, to teach the im portance of preserving the teeth. As to the subject of rubber, to arrive at the discovery of the causes of these troubles from different kind of plates, we must understand all this vast subject of molecules of matter. A plate cannot press anywhere without producing a disease. One may be a poor conductor, or another too good a conductor of either heat or electricity. With strong vital temperaments people may tamper with their digestive apparatus, but the delicate suffer from dyspepsia on the slightest provocation. What is nutrition and weight? His observation is that it depends upon oxygenation; this goes in and seizes upon the living tissues, taking out carbon and hydrogen, and, carrying these out throws them off. To do this the oxygen must be ozone, or in its nascent state, ready to unite with the other elements. The remedy for all disease is first to remove the cause, and if the plate is that cause, take it out.

Dr. Morgan does not profess to understand chemistry to any considerable extent. There are, however, some facts in connection with the use of sulphuric acid. We all know that with living tissues it has a very different action than upon dead. Our surgeons often use this acid for bringing out granulations and restoring lost parts. He thought disease did not come from outside causes alone. If something was taken out of the body, as caloric, we would have disease. He did not know how far magnetism and electricity may affect the system. That the loss of these might affect the health, was possible.

Prof. Taft. The argument of Dr. Morgon, of pathological conditions resulting from the abstraction of caloric, is in favor of Prof. Atkinson's assertion, for this allows the *debris* to remain, and they are certainly foreign matter then. Taking cold is simply holding in and damming back some material which should be thrown out. The pabulum passing in and out of the tissues is essential for the production of heat. By the change of the elements within the body, the force is eliminated which is essential to the condition of health. Motion or movement is a result. It is under the domination of the vital force. Anything arresting or disturbing the conditions of this force aids and produces pathological conditions, whether it comes from within or without. Even a mental condition may produce disease.

Dr. Morgan. Prof. Taft has carried out just the idea which he wished to demonstrate,—the loss of motion or force will cause disease.

Dr. Willis. Much has been said of the manner in which certain functions are performed. But now, why do these changes take place, so as to avoid the rock of which Prof. Atkinson has spoken? The great central force is the sun, and not a single force is in existence which cannot be traced to it. He takes man merely as a machine,—he has nothing to do with his soul. How is the heat in man kept up? Just as in a steam-engine, only that we have an arrangement to carry out the *debris.* The coal contains the force, which was obtained by the vegetation of which it is formed, from the sun. Caloric is not a substance; heat is only a condition.

Prof. Sheppard moved the discussion be closed. Carried.

The report of the committee on Dental Chemistry was then called for.

Dr. Allen. Chairman, reported. The paper related to food. He then said: Let us refer to food, and we find that other nations, who do not change the proportions of natural food as we have done, do not lose their teeth as we do. The Creator has taken the trouble to prepare our foods properly, and we go to work and throw out the mineral portion. 40 lbs. are taken from every barrel of flour, and a child, it is estimated, uses half a barrel per year. Thus it loses 20 lbs. per year, or in 20 years, 400 lbs.: 13, 000 milling establishments and 28, 000 men are employed to do this, while 10, 000 dentists are trying to patch up the loss. The result is that twenty millions of teeth are annually sacrificed, at the nearest estimate. He was pleased to see it proposed to teach children in our schools about the nourishment proper to use. Every old woman knows that her hens require lime to make shells for their eggs.

Prof. Cutler. Children reqnire, in proportion to their weight, more food. His observations have satisfied him that the lacunæ of bone become filled up with the mineral element, making old age and its chilling coldness. The flesh of old age is tougher, more yellow, dense, etc., from its loss of water. Can we extend the life of man,—can we put off the arrival of old age? Can we keep up a normal condition of these bones? If we can take a miserable tuber and cultivate it into a cabbage we can do this. He thought all men suicides who die from old age. We are all such without knowing it. When developing, feed with the mineral, but when the man has matured, crowd him with fine flour. No change takes place in hard tissues after they are once fully formed. In relation 1o the amount of lime, he proposed to use acid fruit, especially if we use limy food, as the oyster, etc. You do not ffnd osseous deposits in the valves of the heart until the bones are filled up first.

Dr. Peebles thought, if he had known it sooner, he wonld have fed his children on bran instead of flour, as it would be cheaper, but he liked to give them as good food as he used. How will we be able to go at this? Can we chenge the whole system of our lives?

Dr. Allen. One remark in reply to Prof. Cutler. In Europe, where the poor do not change the proportions of the food, they have better teeth than the aristocracy. When they come here they do lose their teeth, and why is this? Because they neglect to supply the deficiency of loss. It is well established that the hard tissues do change, and it is essential to keep up the supply for the loss to be replaced. We should populerlize the idea of grinding up the whole grain as the ancients did.

Dr. Dickerman. There seems to be a difference of opinion as to the change of the teeth; may not the climate have something to do with this? He would say that the Graham flour was $1.75 more per barrel, as they cannot cheat as well in the weight as with white flour.

Dr. Allen. In reply to the climate, he would refer to

Humboldt, who says the teeth of Indiaus of all sections were sound. It depends upon the food.

Dr. Allport. We have heard much of late years about proper food. He would not combat the theory, but he felt that the ability of the system to make it up was a matter of importance. He had hardly credited the statement with reference to the health of teeth of peasants abroad. He concluded after examination, that there was not such a difference. In England he thought the teeth worse than in this country; among the nobility good, regular teeth are hardly to be found. The simple idea that they are better is only due to the fact that in Europe they do not think or care for their teeth as we do. It is more due to the intelligence of the people, and their constantly speaking of their teeth, as is done in this country.

Dr. Allen did not consider that Dr. Allport was much amcng the peasantry proper while in Europe. Hack-drivers and waiters live on finer food than the peasant. Many years ago, through the South, he examined the teeth, and found that the field-hands, as a general rule, had good teeth, but the domestics and whites had poor.

Dr. Morgan. Have you any conception of the number examined? How many did you ever see of thirty years of age with sound teeth? He had seen but one of mixed blood who had sound teeth, The negroes are almost universally scrofulous and with unsound teeth.

Dr. Carroll. Having read the paper of Dr. Allen, he used it almost as a text-book, and referred many of his patients to it. He did not think the food alone resided in the flour. The males generally have better teeth than the females. Why? The air breathed has much to do with this. We ignore too often the food taken into our lungs.

Dr. S. A. Chisholm could bring to bear a single experiment. In the inland towns among the Mexicans he found many fine teeth: they lived on rough, plain food. On his return to San Antonio, he found those there had bad teeth. This brought him back to the Harris doctrine,—that as our teeth are developed so they remain. Cleanliness, etc. are necessary for healthy teeth.

Prof. J. S. Knapp. Should we discuss so wide a sphere?

Prof. Stellwagen said that, after reading Dr. Allen's report, he had become more impressed with the truth of the theory. What can be plainer than the proposition that, if the elements necessary for the formation of the dental structures are not introduced into the system, we cannot expect to find well-developed and normal organs?—or can we better select the proportions of our food than has been already done for us by nature?

With his own family he had experienced some of the difficulty spoken of in introducing the use of the bran bread, but he thought he had seen the benefit of it. He also laid great stress upon the importance of keeping the saliva in an alkaline condition. This, if it only prevents the acid action between the teeth, does well; but he was almost ready to search for direct osmosis between the fluids of the pulp and tubuli and mouth. The practice of this is simple enough consistimg merely in rubbing *prepared* chalk around the mouth pressing it between the teeth upon retiring, and washing out thoroughly, two or three times every day, with lime water.

His first child, a daughter, erupted most of her teeth prematurely, her second deciduous molars coming through when she was nineteen months old; the inferior were so defective that he was forced to fill one with gold, and the other (from the close proximity of the disease to the pulp, leaving but a thin septum of dentine intervening, so that it could not withstand the pressure for consolidating the metal) with amalgam. There were several other small cavities left at the time. Dreading to operate, he had postponed filling until the child was thirty-one months of age, when he had excavated one which presented the worst appearance, to satisfy himself if there was much softening under the enamel, and he found no more than he considered an average amount, but felt that the tooth up to that time was very little, if any, worse than when the first fillings had been inserted, *ten* months before. During this time he had had the child's mouth treated with the chalk and lime-water. Bread made from flour that had not been robbed of its bran was eaten very freely, but not exclusively, every day in the week. In this way he thought that kind of bread was very palatable and appetizing.

The cow's milk used by his children,—for they both had to depend mostly upon artificial food,—after being properly diluted with water, to prevent overtaxing their digestive powers, or rather to give it about the same proportion of water as in human milk, was sweetened, and to every half pint one teaspoonful of lime water was added. His second child, a daughter also, had been fed with milk prepared in this way from the time she was ten months old, and her teeth were much better in appearance and texture. He attributed it to the difference in the diet and the use of antacids.

Plenty of good air and sunlight were of course indispensible for all persons having diatheses favoring caries; the chemical and vital forces must be kept up by free supplies of their correlative forces, heat, light, electricity, etc. For this purpose he advised all his patients to take regular walks on the sunny side of the street; children he preferred to have spend as much as possible of their time in the open and direct rays of the sun.

In treating teeth, a dentist should never forget that they are part and parcel of the whole economy; if diseased, the vital force of the individual must be cared for, and, if necessary, his system elevated to the proper standard by attention to open air exercises in the sunlight, food, and these sometimes assisted by the materia medica. In many cases he had refused to operate until the general health was restored; found patients thus treated were better able to stand the pain; presented mouths whose secretions were nearer the normal condition, and, consequently, the fillings could be very naturally expected to remain intact.

He hoped others present would give their experience with the chalk, lime water, bran bread, etc., in a word, the treatment spoken of. He felt satisfied that it could do no harm, and in some fifty cases, where pretty thoroughly tried, the condition of the teeth was materially improved, the dentine in some becoming harder, and less frequently attacked by caries. In this way he hoped the profession would make a material steps towards improving these organs in coming generations as well as the present one, and thus hasten the millennium when dentists and physicians should no longer be required.

*To be Continued.*

